# The electronic structure of metal oxide/organo metal halide perovskite junctions in perovskite based solar cells

**DOI:** 10.1038/srep08704

**Published:** 2015-03-03

**Authors:** Alex Dymshits, Alex Henning, Gideon Segev, Yossi Rosenwaks, Lioz Etgar

**Affiliations:** 1 The Hebrew University of Jerusalem, Institute of Chemistry, Casali Center for Applied Chemistry, Jerusalem 91904, Israel; 2 Department of Physical Electronics, School of Electrical Engineering, Tel Aviv University, Ramat Aviv 69978, Israel

## Abstract

Cross-sections of a hole-conductor-free CH_3_NH_3_PbI_3_ perovskite solar cell were characterized with Kelvin probe force microscopy. A depletion region width of about 45 nm was determined from the measured potential profiles at the interface between CH_3_NH_3_PbI_3_ and nanocrystalline TiO_2_, whereas a negligible depletion was measured at the CH_3_NH_3_PbI_3_/Al_2_O_3_ interface. A complete solar cell can be realized with the CH_3_NH_3_PbI_3_ that functions both as light harvester and hole conductor in combination with a metal oxide. The band diagrams were estimated from the measured potential profile at the interfaces, and are critical findings for a better understanding and further improvement of perovskite based solar cells.

Organo lead halide perovskite is a promising material in photovoltaic solar cell research. During the past five years, the power conversion efficiency of perovskite based solar cells drastically increased, exceeding energy conversion efficiencies of 20.1%^1^,^2^,^5^,^8^. A long diffusion length of electrons and holes was recently observed in organo metal halide perovskite and is one of the main reasons for the high solar cell efficiency^3^,^4^. Several techniques are used for the perovskite deposition including solution-processed (one-step and two-step) deposition, evaporation method, and vapor assisted solution process (VASP)^5^,^6^,^7^,^8^,^9^. Solar cells with a high open circuit voltage of more than 1.3 V were demonstrated using CH_3_NH_3_PbBr_3 _as the light harvester^10^,^11^.

Despite the rapid increase in the photovoltaic performance of perovskite based solar cells, the operation mechanism of the metal oxide/perovskite junction is still under debate. In a recent work, the junctions of a CH_3_NH_3_PbI_3−x_Cl_x_ based solar cell were investigated by electron beam-induced current (EBIC) and a *p-i-n* junction was suggested^12^. An indication for carrier accumulation in the perovskite absorber material was observed by impedance spectroscopy measurements^13^. Kelvin probe force microscopy (KPFM), a scanning force microscopy based technique, was recently used to characterize the perovskite top layer^14^,^15^ where the presence of a small potential barrier at the grain boundaries (GBs) was found. Bergmann *et al.* have recently measured cross-sections of a complete perovskite based solar cell under illumination with KPFM and showed that the potential is similar to a *p-i-n* type junction^16^. KPFM is based on measuring and compensating the electrostatic forces between a sample and an AFM tip to obtain the contact potential difference (CPD)^17^,^18^, which is a measure of the Fermi level energy if the electron affinity is known.

In this work, we use KPFM to measure the potential along the metal oxide/CH_3_NH_3_PbI_3_ interface for two different nanocrystalline (nc) metal oxides, TiO_2_ which functions as an electron acceptor due to the possibility for electron injection, and porous Al_2_O_3_ which acts as a scaffold^19^. In the current solar cell configuration the CH_3_NH_3_PbI_3_ functions both as a light harvester and a hole conductor, therefore the solar cell operates without an additional hole transporting material. The chosen device structure facilitates the preparation of smooth cross-sections and simplifies the KPFM measurement and its interpretation. Our high-resolution KPFM measurements in the dark provide insight into the interface electronic structure. A surface space charge region of ca. 45 nm is determined for the nc-TiO_2_/Perovskite junction and is expected to enhance charge carrier separation and solar cell performance. The free carrier density was estimated from the band bending at the perovskite grain boundaries induced space charge region.

## Results and Discussion

**Figure 1:**
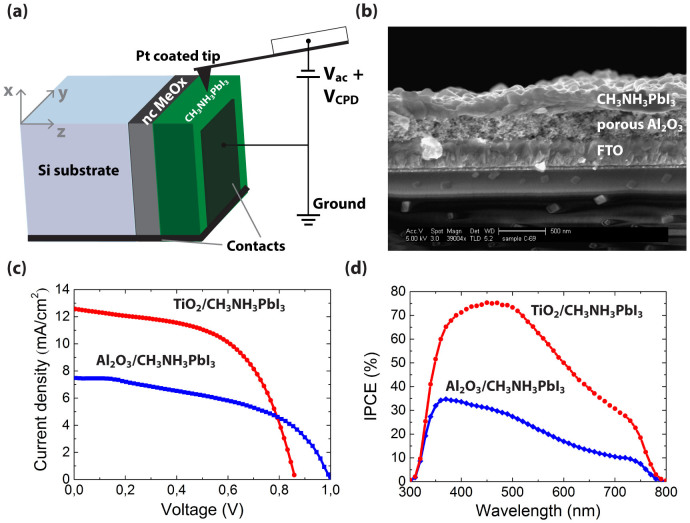
(a) Schematic illustration of the KPFM measurement setup. The contact potential difference (CPD) is determined by compensating the electrostatic forces between the tip and sample where nc MeOx stands for nanocrystalline metal oxide. (b) HR-SEM images of the hole-conductor-free nc-Al_2_O_3_/MAPbI_3_ perovskite solar cell.(c) Current-voltage curves of the studied solar cells and (d) the corresponding IPCE spectra.

Kelvin probe force microscopy on cross-sections of hole-conductor-free perovskite based solar cell in dark (Fig. 1a) was used to determine the electronic structure of the interfaces that are critical for the performance of these solar cells. Perovskite solar cell cross-sections were prepared in a nitrogen glovebox by cleavage. Capillary forces were minimized allowing a small tip-surface distance hence better lateral resolution compared to an AFM setup in ambient. A typical structure of the hole-conductor-free perovskite solar cell is shown in the high resolution scanning electron microscopy (HR-SEM) micrograph (Fig. 1b). The MAPbI_3_ perovskite is deposited on top of the metal oxide using the two-step deposition process described earlier^6^,^20^. Table 1 and figure 1c show the photovoltaic parameters and the current voltage curves of the hole-conductor-free MAPbI_3_ (CH_3_NH_3_ = MA) solar cells using nc-TiO_2_ or nc-Al_2_O_3_ as the metal oxide. Figure 1d presents the incident photon to current efficiency (IPCE) of the corresponding cells. For both cells, the response covers the whole visible range. However, for the nc-TiO_2_/MAPbI_3_ cell, the IPCE reaches 75%, while for the nc-Al_2_O_3_/MAPbI_3_ cell, 35% IPCE is achieved in good agreement with the current density-voltage (J-V) characteristics measured under a solar simulator. Upon visible-light excitation of the TiO_2_/MAPbI_3_/Au cell, electrons and holes are generated inside the perovskite layer. The electrons are then injected into the conduction band of the TiO_2_ and driven towards the FTO, whereas holes are transported through the perovskite layer to the Au contact.

For the Al_2_O_3_/MAPbI_3_/Au configuration, injection of electrons into the nanoporous Al_2_O_3_ is energetically not allowed; therefore, the photogenerated charge carriers are transported through the MAPbI_3_ film to the appropriate contacts.

**Figure 2:**
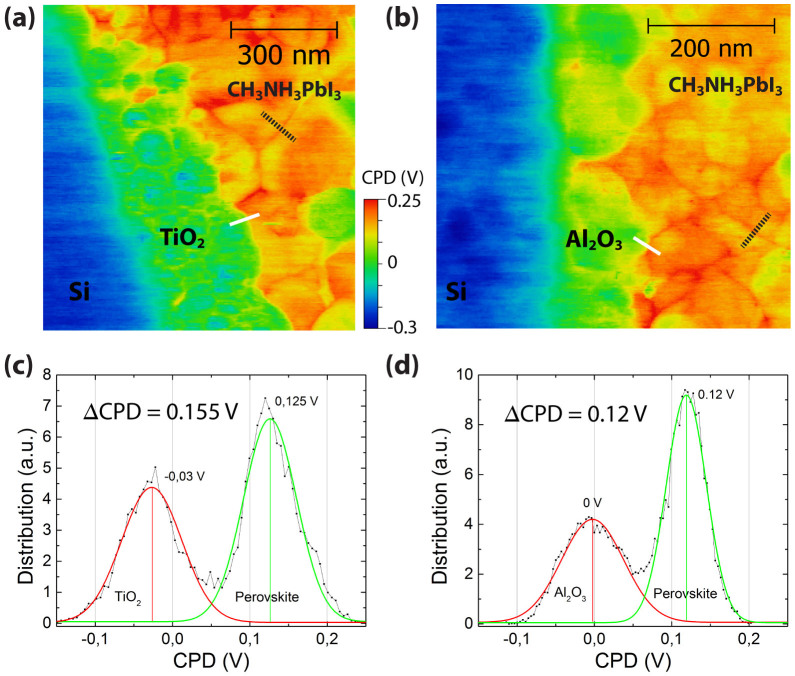
KPFM images of solar cell cross-sections showing (a) the nc-TiO_2_/MAPbI_3_ and (b) the nc-Al_2_O_3_/MAPbI_3_ interface.The white line indicates the CPD profiles shown in figure 4. (c) CPD statistical distribution for the CPD image of the nc-TiO_2_/MAPbI_3_ interface (d) CPD statistical distribution for the CPD image at the Al_2_O_3_/MAPbI_3_ interface. The dashed line indicates the CPD profile along a grain boundary shown in figure 3b.

The KPFM cross-section images of the two different cells in dark, nc-TiO_2_/MAPbI_3_ and the nc-Al_2_O_3_/MAPbI_3_, are presented in Figure 2a and 2b. The high spatial resolution was achieved by maintaining the tip-sample distance below 5 nm at a slow scan rate of 0.1 Hz. The perovskite penetrates through the nc-Al_2_O_3_, sintered to the grounded contact; consequently, the Fermi level is assumed to be aligned and equal in all three layers. This allows a quantitative interpretation of the measured potential for both solar cell structures. Figure 2c and 2d show the CPD statistical distribution for the two interfaces; A CPD variation of 90 mV was determined for both metal oxides and a CPD variation of 65 mV was found for the perovskite layer. These variations are mainly due to the nanostructure and inhomogeneous surface of the measured materials entail a high amount of defect states. The measured average potential difference, ΔCPD, at the interface between nc-TiO_2_ and MAPbI_3_, as well as between the nc-Al_2_O_3 _and MAPbI_3_ interface is 0.155 V and 0.12 V, respectively. Although the nc-TiO_2_/MAPbI_3_ based solar cell has a higher efficiency, the potential difference between the two interfaces in the dark (35 mV) is negligible small. Some of the perovskite grains have a lower CPD and are expected to be less active in the solar cell, as previously observed with EBIC measurements^15^.

**Figure 3:**
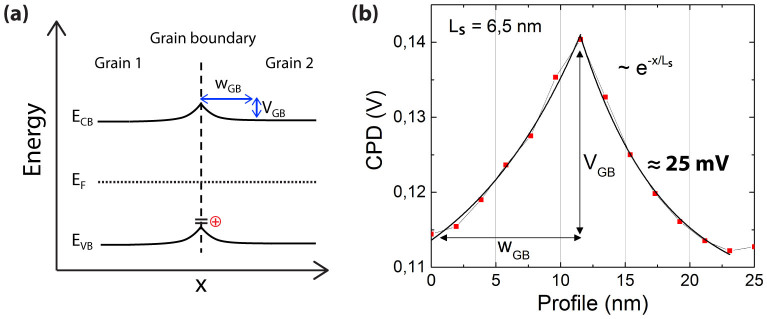
(a) Electronic band structure at the grain boundaries where the potential is slightly higher (more p-type) compared with the grain body due to hole traps. V_GB_-potential height at the grain boundaries. W_GB_-width of potential barrier. (b) CPD profile along the grain boundary between two adjacent perovskite grains indicated in the CPD image of figure 2a.

The CPD is higher (more positive) at the grain boundaries between adjacent perovskite crystals by ca. 25 mV (see figure 3b), implying a minor effect on the perovskite solar cell performance as this potential barrier is around the thermal voltage at room temperature. Figure 3a shows a schematic band diagram for the case of positive charge accumulation (hole traps) at the grain boundary. It is possible to calculate the width of the barrier potential at the grain boundaries using known barrier models^21^,^22^ provided the grain boundary is of negligible width compared to the grain size. This model was used to analyze grain boundaries in p-type polycrystalline silicon, II-VI semiconductors and chalcopyrite materials.

The electrostatic screening length, L_S_ (within Ls charges are electrostatically screened; beyond this length charges are not affected by other charges) is extracted by fitting the measured potential profile across the GB (Fig. 3b) with an exponential function and is approximately 6.5 nm, which is much smaller than the average grain size of the perovskite (grains of around 200 nm can be observed in figures 2a and 2b). The dopant concentration, N_d_, can be estimated with equation 1^22^,
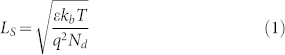
where ε = ε_0_ε_p_ (ε_p_ ≈ 20^23^ is the dielectric constant of the perovskite and ε_0_ is the permittivity), k_b_ is the Boltzmann constant, T is the temperature and q is the elementary charge. By using extracted L_S_ in equation 1, the dopant density (N_d_) was estimated to be 7 × 10^17^ cm^−3^, which is in a good agreement with reported values observed by Guerrero *et al.*^24^

**Figure 4:**
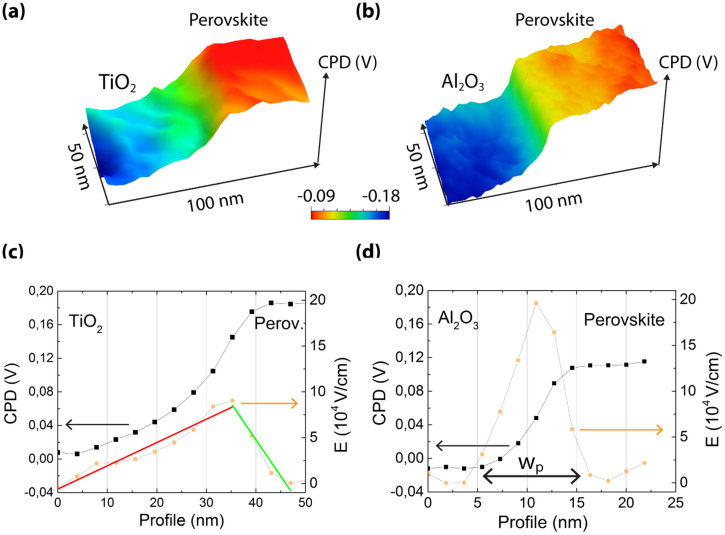
(a) Three dimensional representation of the measured CPD and the nc-TiO_2_/MAPbI_3_ and (b) the nc-Al_2_O_3_/MAPbI_3_ interface. (c) CPD profile and the corresponding electric field, dCPD/dx, for the nc-TiO_2_/MAPbI_3_ (d) for the nc-Al_2_O_3_/MAPbI_3_ interface. The depletion width at the perovskite is defined as W_p_.

The CPD profiles of the metal oxide/MAPbI_3_ interfaces, shown in figures 4a–d, provide estimation for the junction depletion width. Figure 4c and 4d show the CPD and the electric field, E = dCPD/dx, across the interface for the nc-TiO_2_/MAPbI_3_ and the nc-Al_2_O_3_/MAPbI_3_ cell structure, respectively. Accordingly, the depletion width for the nc-TiO_2_/MAPbI_3_ heterojunction is ≈45 nm with a maximum electric field of E ≈ 9·10^4^ V/cm. In the case of the nc-Al_2_O_3_/MAPbI_3_ interface, there is no electron injection from the MAPbI_3_ into the nc-Al_2_O_3_, since it functions rather as a scaffold. The electric field profile for the nc-Al_2_O_3_/MAPbI_3_ interface is narrower, showing a depletion (space charge region) width of around W_p_ ≈ 10 nm in the perovskite side of the junction (figure 4d). This corresponds to the depletion region width at the perovskite side at the nc-TiO_2_/MAPbI_3_ interface.

**Figure 5:**
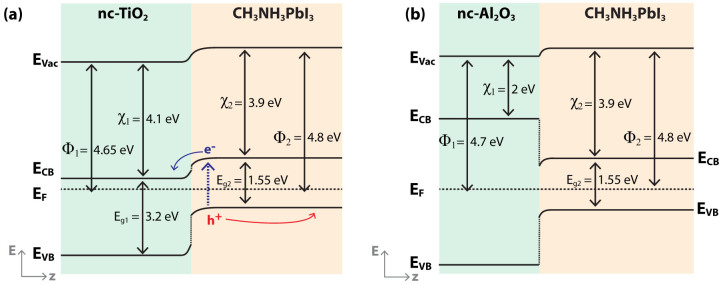
(a) and (b) Band diagram of the TiO_2_/MAPbI_3_ and Al_2_O_3_/MAPbI_3_ interfaces, respectively, calculated using the KPFM measurements. The conduction and valence bands position are according to Ref. 18 and 26.

Figures 5a and 5b show the suggested energy band diagrams for the nc-TiO_2_/MAPbI_3_ and the nc-Al_2_O_3_/MAPbI_3_ interfaces using the measured work functions in dark. As opposed to the nc-Al_2_O_3_, the nc-TiO_2_ has an active role in the electron transport. For the nc-TiO_2_/MAPbI_3_ interface (Fig. 5a) electron transport takes place mainly through the nc-TiO_2_ since the electron injection is favorable. The measured space charge region at this interface assists in the charge separation and inhibits recombination which assist to the PV performance.

For the nc-Al_2_O_3_/MAPbI_3_ interface, an oxide-semiconductor band structure is proposed with a relatively small band bending at the perovskite side. Since the 80 nm thin Al_2_O_3_ layer is nanoporous, the perovskite penetrates through the Al_2_O_3_ enabling electron transport to the contacts. We note that experiments with an Al_2_O_3_ layer thickness >80 nm drastically reduced the solar cell performance.

The long diffusion length assists in the charges transport to the corresponding contacts.

Table 2 summarizes the parameters obtained in this study. The carrier density of the perovskite was calculated using the electronic barrier model; the depletion region width and the electric field were estimated from the cross section KPFM measurements.

## Conclusions

Cross-sections of hole-conductor-free perovskite solar cells were measured using KPFM. The measured potential differences between the nanoporous metal oxide and the perovskite in dark are consistent for both nc-TiO_2_ (ΔCPD = 0.155 V) and nc-Al_2_O_3_ (ΔCPD = 0.12 V) based cells and were used to estimate the band diagrams. Moreover, the measured width (w_p_ = 10 nm) of the depletion region at the perovskite side is the same. However, while the measured band bending at the nc-Al_2_O_3_/MAPbI_3_ side is negligible small, we measured a 45 nm depletion region width at the TiO_2_ side of the nc-TiO_2_/MAPbI_3_ interface. This depletion region contributes to the separation of photogenerated charge carriers and thus a higher performance of the TiO_2_ based cells. The measured potential difference of 25 mV at the perovskite grain boundaries reveals hole accumulation at the GBs and plays a minor role for the solar cell performance. The results shed additional light on the electronic structure of these highly efficient perovskite based solar cells.

## Experimental

### Material synthesis

TiO_2_ paste DSL 90-T composed of 20 nm particles was purchased from DYESOL. The TiO_2_ paste was diluted with ethanol in ratio of 1:4 by weight and deposited by spin coating at 2000 r.p.m. for 10 sec. The TiO_2_ film was annealed at 500°C for 30 min. The nc-Al_2_O_3_ 20 wt.% in isopropanol (<50 nm particle size) was purchased from Sigma-Aldrich. The nc-Al_2_O_3_ was diluted in isopropanol in ratio of 1:12 by weight. The deposition and the annealing conditions were the same as for TiO_2_.

CH_3_NH_3_I was synthesized as described previously^25^, by reacting 30 mL of methylamine (40% in methanol, TCI) and 32.3 mL of hydroiodic acid (57 wt% in water, Aldrich) in a 250 mL round bottom flask at 0°C for 2 h with stirring. The precipitate was recovered by putting the solution on a rotavap and carefully removing the solvents at 50°C. The yellowish raw product of methylammonium iodide (CH_3_NH_3_I) was washed with ethanol by stirring the mixture for 30 min. Then the mixture was filtered and washed three times with diethylether. After filtration, the solid was collected and dried at 70°C in a vacuum oven for 24 h.

### Device fabrication

The substrate of the device was a SNO_2_:F (FTO) conducting glass (15 Ω·cm^−1^), Pilkington). A blocking layer was deposited on the FTO glass using a solution of titanium diisopropoxidebis(acetylacetonate) (TiDIP, 75% in isopropanol, Aldrich) in ethanol. The TiDIP solution was spin coated and then annealed at 450°C for 35 min. The TiO_2_ solution or the Al_2_O_3_ solution were spin coated and annealed at 500°C for 30 min subsequent to TiCl_4_ treatment for 30 min at 70°C and annealing at 500°C for 30 min.

The synthesis of the CH_3_NH_3_PbI_3_ on the TiO_2_ surface was carried out by a two-step deposition technique.

First, PbI_2_ was dissolved in DMF and dropped onto the TiO_2_ film and spin coated, followed by annealing at 70°C for 30 min. In the second step, the cell was dipped into methylammonium solution. Following the dipping step, the samples were annealed at 70°C for another 30 min. Finally, the back contact was deposited by evaporating 50 nm of gold under pressure of 5*10^−6^Torr. The active area was 0.09 cm^2^.

The KPFM cross sections samples were prepared on a crystalline silicon substrate. The Si wafer was etched from native oxide by hydrofluoric acid (48 wt% in water, Aldrich) for 15 min and cleaned by oxygen plasma for 1 min.

### Photovoltaic characterization

Photovoltaic measurements were made on a New Port system, composed of an Oriel I–V test station using an Oriel Sol3A simulator. The solar simulator is class AAA for spectral performance, uniformity of irradiance, and temporal stability. The solar simulator is equipped with a 450 W xenon lamp. The output power is adjusted to match AM1.5 global sunlight (100 mWcm^−2^). The spectral match classifications are IEC60904-9 2007, JIC C 8912, and ASTM E927-05. I-V curves were obtained by applying an external bias to the cell and measuring the generated photocurrent with a Keithley model 2400 digital source meter. The voltage step and delay time of photocurrent were 10 mV and 40 ms, respectively. Oriel IQE-200 was used to determine the monochromatic incident photon-to-electric current conversion efficiency. Under full computer control, light from a 150 W xenon arc lamp was focused through a monochromator in the 300–1800 nm wavelength range onto the photovoltaic cell under test. The monochromator was incremented through the visible spectrum to generate the IPCE (λ) as defined by IPCE (λ) = 12,400 (Jsc/λ φ), where λ is the wavelength, Jsc is the short-circuit photocurrent density (mA cm^−2^), and φ is the incident radiative flux (mWcm^−2^). Photovoltaic performance was measured by using a metal mask with an aperture area of 0.09 cm^2^.

### High Resolution Scanning Electron Microscopy (HR-SEM)

SIRIONHR SEM was performed with FEI (Field Emission Instruments), The Netherlands. The measurement conditions were 5 kV at a magnification of 39,000.

### Kelvin probe force microscopy (KPFM)

Various locations of the cleaved cross-sections were measured by KPFM and consistent CPD profiles with same variations were obtained for each solar cell type. The measured CPD was constant over a day range. Topography and CPD were measured with a slow scan rate (0.1 Hz) and in the attractive force regime (force-distance curve) in order to avoid tip-sample crashes. Amplitude modulation KPFM was carried out with a commercial AFM (Dimension Edge, Bruker Inc.) inside a nitrogen glove box with less than 1 ppm H_2_O at room temperature. The CPD was measured simultaneously with the topographic signal at an effective tip sample distance of 5 nm during scanning. The topographic height was obtained by maintaining the amplitude of the first cantilever resonance (f_1st_ ≈ 75 kHz) at a predefined amplitude set point of approximately 10 nm. The CPD was determined by compensating the ac component of the electrostatic force at angular frequency ω with an applied dc voltage ( = |CPD|) in a feedback control loop. To separate topographic from CPD signal, increase the sensitivity, and minimize probe-sample convolution effects, the AC electrostatic force component was generated at the second resonance^26^, f_2nd_ ≈ 450 kHz, of the cantilever by applying an ac voltage of about 500 mV. Highly conductive cantilevers with Pt/Ir coating (PPP EFM, Nanosensors) were used for KPFM.

## Author Contributions

A.D. prepared the cells and characterized them, analyzed the data and took part in the discussion. A.H. performed the KPFM measurements and took part in the discussion and the preparation of the paper. Y.R. is the supervisor of A.H. and he participated in the discussion. L.E. is A.D.'s supervisor; L.E. wrote the paper and planned its experiments. G.S. did the simulation of the band diagram and contribute in the discussion part. The paper was edited and approved by all authors.

## Figures and Tables

**Table 1:** Photovoltaic parameters of the studied hole-conductor-free perovskite solar cells

	Jsc (mA/cm^2^)	FF	Voc (V)	Efficiency (%)
nc-TiO_2_/MAPbI_3_	12.56	56	0.86	6.1
nc-Al_2_O_3_/MAPbI_3_	7.25	48.6	1.0	3.9

**Table 2:** Depletion region width, carrier density and maximum electric field at the junction

Sample	Depletion width Wp/Wn (nm)	N_d (Perovskite)_ (cm^−3^) (calculated from the grain boundaries)	ΔCPD (V)	Electric field max. (V/m)
nc-TiO_2_/MAPbI_3_	10/35	7 × 10^17^	0.155	9 × 10^6^
nc-Al_2_O_3_/MAPbI_3_	Wp ≈ 10	7 × 10^16^	0.12	*
